# Expediting the Implementation of Closed-Loop Supply Chain Management: a Facilitated Case Study on Re-using Timber in Construction Projects

**DOI:** 10.1007/s43615-022-00186-6

**Published:** 2022-07-04

**Authors:** Jannie Coenen, Rob E. C. M. van der Heijden, Allard C. R. van Riel

**Affiliations:** 1 Institute for Management Research (IMR), Radboud University (RU), PO Box 9108, NL-6500 HK, Nijmegen, the Netherlands; 2 Faculty of Business Economics, Hasselt University, Martelarenlaan 42, B-3500 Hasselt, Belgium

**Keywords:** Closed-loop supply chain management, Dynamic complexity, Deep uncertainty, Systematic capability maturity approach, Dutch construction supply chain, Circular economy

## Abstract

An increasing number of firms are aiming to implement closed-loop supply chain (CLSC) management to contribute to a more circular economy. However, for many of these firms, it is difficult to translate this strategic aim into fruitful operational decisions. They need to address many deep uncertainties and dynamic complexities in their supply chain system, which make their transition towards CLSC management challenging. This article aims to develop a better understanding of how supply chain actors taking steps towards CLSC management could be supported to reach higher levels of maturity in dealing with deep uncertainty and dynamic complexity. This is investigated in a single, facilitated, embedded case study: a future-oriented decision-making process regarding the use of timber with four real-world actors in the construction industry. The process is structured and supported with analyses, following a methodology based on the capability maturity approach. In this empirical context, the selected approach is shown to have positive effects on clarifying the potential impact of transitions to CLSC management. Furthermore, it stimulates important learning processes during the transition, and as such supports actors to achieve higher levels of maturity and to take further steps towards CLSC management. In this context, a conceptual distinction is made between ‘situational maturity’ and ‘mental maturity’, which enriches double-loop learning theory in the context of transitions.

## Introduction

**Fig. 1:**
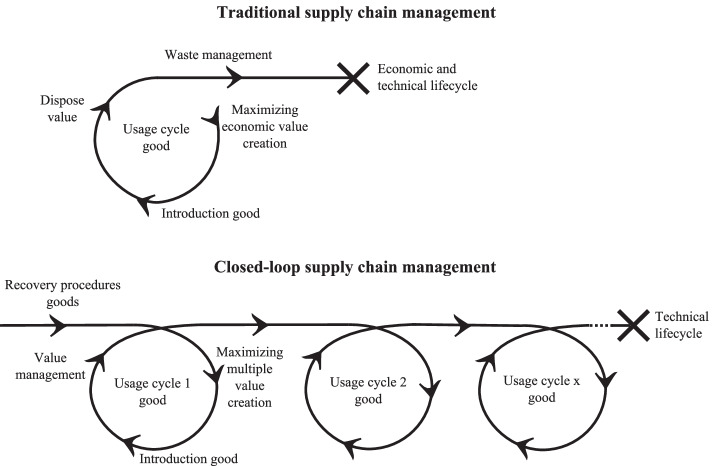
Traditional supply chain management versus CLSC management

In recent years, a government-supported programme has been deployed in the Netherlands, aimed at developing a more circular economy [1]. The underlying discourse argues that there is a need for radical changes in production-consumption cycles, aimed at re-using resources and products, to reduce their global carbon footprint. The discourse triggers a gradually increasing number of initiatives from business to focus on closing the loops in their supply chain. Such initiatives become manifest in markets where new technologies are combined with new principles of product design, remanufacturing, service organization, and supply chain collaboration [e.g. 2]. A closed-loop supply chain (CLSC) differs fundamentally from a traditional supply chain. The latter is based on a single ‘take-make-use-dispose’ cycle with the aim of maximizing the economic value of a good over that cycle (see Fig. 1). In contrast, a CLSC maximizes value in multiple dimensions (notably the well-known triple P: people, planet, and profit) over the technical lifecycle of a good [e.g. 3]. The dynamic recovery of value in sequential usage cycles of a good or its components plays a crucial role in a CLSC [2, 4].

Achieving CLSC management requires a structural change in the way supply chain actors think and act. Although a growing number of supply chain actors participate in the discourse on the circular economy, so far successful breakthroughs towards CLSC management are rare [5]. CLSC experiments and projects occur at present mostly in protected bubbles, embedded within a wider landscape that is dominated by business-as-usual (BAU) practices. The intended, more encompassing changes in the structure and operation of supply chains require a transition. This transition triggers supply chain actors (SCAs) to answer questions such as which changes are crucial, how could these changes influence supply chain dynamics, and how could they affect the businesses of each of the actors involved?

Trying to answer such questions helps to unfreeze the dominant way of thinking and acting of SCAs and to make a fundamental shift towards a new way of thinking and acting [6, 7] and hence to make a mental shift [8]. To achieve such a fundamental shift, ‘double-loop learning’ of the involved SCAs has been argued to be crucial [9]. Accordingly, SCAs must learn to view the system they are part of in a fundamentally different way and to make trade offs between various interests in a very different way than what they were used to. Double-loop learning should therefore be stimulated in SCAs that aim to implement CLSC management, both at the individual actor level and collectively within the network of collaborating actors [e.g. 10, 11].

Recent studies illustrate that insufficient attention has been paid to such learning processes in the context of sustainability transitions [12]. CLSC management studies argue that double-loop learning should particularly focus on developing the capability to deal with deep uncertainty and dynamic complexity [4, 10]. Other studies argue that the occurrence and potential effects of those phenomena hinder SCAs to change their BAU approach [11, 13]. Deep uncertainty exists notably when decision-makers do not know, or cannot agree upon, among other things, how likely it is that, and to what extent, different future scenarios would affect their businesses [14, 15]. Deep uncertainty is strongly related to behaviour of complex systems (e.g. exponential growth or decay, oscillation), that results from dynamic co-existing causal interactions between the system and its environment and feedback mechanisms within the system [16, 17]. Deep uncertainty and dynamic complexity may drive actors apart and/or lead to a wait-and-see attitude of risk-aversive actors towards structural system change [18]. Alternatively, it may trigger higher levels of collaboration, knowledge gathering, and/or actions based on trial and error by actors with a more risk-taking attitude [19]. Both types of actors need to collaborate in a supply chain, however, because their business activities are interdependent. Therefore, it is important for them to find a shared strategy for transitioning towards a CLSC. A problem is that few, if any, CLSC management studies offer well elaborated and validated approaches for supporting SCAs to address these issues in a systematic and integrated way [4].

This article addresses two knowledge gaps. The first gap concerns the lack of a clear understanding of how deep uncertainty and dynamic complexity affect SCAs’ decision-making processes in a transition towards CLSC management. The second gap concerns the lack of insight into how SCAs’ double-loop learning, and maturity development can be systematically stimulated to support them in dealing with deep uncertainty and dynamic complexity. Both gaps were addressed from a theoretical perspective in [10]. These authors presented a stepwise analytical and process-based approach (hereafter referred to as ‘systematic capability maturity approach’). The assumption underlying this approach is that when SCAs develop a joint strategy to implement CLSC management, they experience a clear need for grasping the many uncertainties related to this structural change in their ways of working. Moreover, it is hypothesized that when SCAs apply the approach, they learn to deal in a ‘more mature’ way with raising deep uncertainty and dynamic complexity issues in their system when preparing for making an operational transition towards CLSC management. However, because this approach did not go beyond this theoretical claim yet, its application in a real-world empirical setting is considered essential for underpinning this claim. The research question is therefore: *How can a systematic capability maturity approach expedite the attainment of higher levels of maturity of SCAs who prepare for implementing CLSC management?*

To address this research question, a facilitated case study with real-world SCAs was conducted in the period between June 2019 and November 2020, focusing on the reuse of timber in a Dutch construction supply chain. The present article reports on this case study. The “Theoretical Background of the Systematic Capability Maturity Approach” section discusses the theoretical background and characteristics of the applied approach in more detail. The “Case Study Design” section elaborates on the facilitated case study. The “Findings” section presents the findings of the study, whereas the “Discussion” section discusses these findings and avenues for future research. The “Conclusion and Limitations” section presents the conclusions and limitations of the study.

## Theoretical Background of the Systematic Capability Maturity Approach

**Fig. 2:**
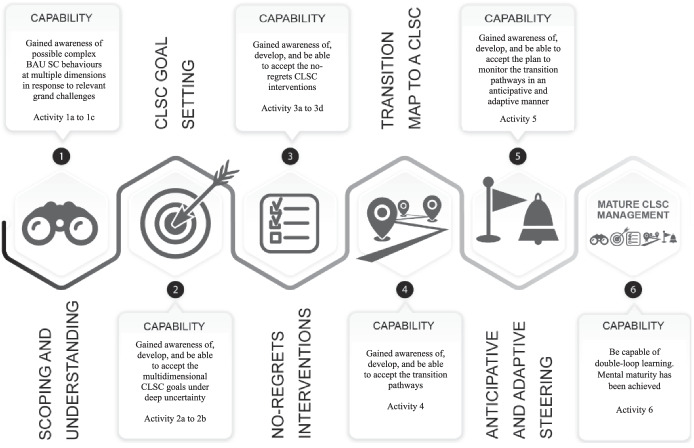
Conceptual framework of the CLICK methodology [10]

The systematic capability maturity approach mentioned above was elaborated under the label ‘CLICK methodology’ (‘Closed-Loop Integration: Collective Keystones’) [10]. The approach consists of a process-oriented conceptual framework and a toolkit with analytical and collaborative methods. The framework is inspired by the concept of ‘capability maturity growth’ that describes the gradual development of a variety of management capabilities of actors involved in a (decision-making) process [20]. The CLICK framework assumes stepwise double-loop learning, starting with an awareness of grand challenges until having fully internalized CLSC thinking and acting under deep uncertainty and dynamic complexity in the core business. The toolkit aims to support SCAs to go through the process expressed in the framework and to stepwise develop capabilities and a higher level of maturity in dealing with the challenges of CLSC management. Figure 2 visualizes the framework in terms of the six stages of maturity and related capabilities. In Maturity Stage 6, the involved SCAs apply mature CLSC management practices structurally, implying that all capabilities related to the different maturity stages are internalized and transformed into a fundamentally new way of thinking and acting at individual and collective level in support of CLSC management. This process has been labelled as achieving mental maturity. Achieving mental maturity in CLSC management assumes a fundamental change in perception, beliefs, and core values of the involved actors. It results in a mind-set, shared by all SCAs, that structurally breaks away from the dominant probabilistic, often one-dimensional, short-term, and static approach focused on economic value, that presently characterizes traditional supply chain management, towards an exploratory, multidimensional, and dynamic approach [10, 16].

An important nuance must be made here, inspired by literature on situational maturity [21, 22]. This literature argues that differences in context and activities affect how managers think. Linking this insight to the concept of double-loop learning, in this study, *situational maturity* is used to refer to an actor’s process of double-loop learning connected to and influenced by the characteristics of the unique context that is created to stimulate the learning process. An actor may develop situational maturity by means of learning within the boundaries of that unique context, while at the same time not being capable of translating that situational way of thinking and acting into an overarching strategy for the whole business based on the same principles. In that case, the learning effects in the unique context do not cross the boundaries of that context towards, e.g. the core business. This could result in continuation of the BAU. Double-loop learning and attaining situational maturity by an actor in a unique context are therefore not a guarantee for the attainment of *mental maturity* by that actor. However, it can function as one of the steppingstones for that actor towards the attainment of mental maturity. The conceptual difference between situational and mental maturity is not explicitly addressed in double-loop learning literature, but it is relevant for stimulating and empirically studying maturity development of SCAs towards CLSC management.

**Fig. 3:**
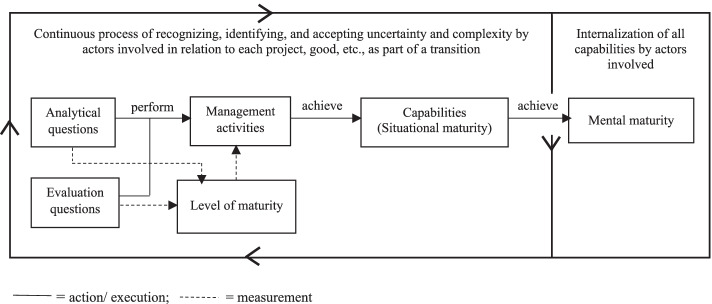
Systematic capability maturity approach

In line with the concept of capability maturity growth, each maturity stage of the CLICK methodology assumes that a *capability* is developed by SCAs to deal with deep uncertainty and dynamic complexity in the context of that specific stage. To develop a capability, the SCAs need to successfully perform specific *management activities*. Whether or not a SCA is able to perform each of these management activities successfully depends on the *level of maturity* achieved in relation to that specific activity. A management activity can be performed at increasing levels of maturity, defined as follows. Maturity Level 1: the SCA starts to become aware of deep uncertainty and/or dynamic complexity related to the specific management activity. Maturity Level 2: the SCA is fully aware of and in addition able to deal with ‘known unknowns’ and/or dynamic complexity. Maturity Level 3: the SCA agrees or agrees to disagree with (the results of) collective decisions made by the network of involved SCAs in relation to the specific management activity. Measuring the maturity levels achieved by the individual SCAs requires answers to a set of specific *analytical and evaluation questions* regarding the way the SCAs execute the management activities. If all management activities related to a specific capability are performed successfully, the capability’s maturity level has been achieved. Figure 3 depicts the systematic capability maturity approach. The operationalisation of capabilities, related management activities, maturity levels, and their measurement in the case study are presented in the next section.

The application of the capability maturity approach in practice assumes an active role of an independent researcher. On the one hand, the researcher designs an interactive process with the involved SCAs, based on formulating questions, providing essential data and information, and collaboratively processing the information exchange into a shared narrative aimed at making further steps towards CLSC management. On the other hand, the researcher makes informative analyses, collects the arguments in the debate among the SCAs and the decisions they make, and analyses the patterns in these data, e.g. the level of maturity that is reached. The relevance of the researcher’s roles has been emphasized since the first research on decision support systems [23], which at that time was characterized as chauffeuring the participatory decision-making process. More recently, the term ‘facilitated modelling’ is used [e.g. 24], notably linked to case-based participatory decision-making contexts with selected stakeholders. The design of the facilitated case study initiated to answer the research question posed in the “Introduction” section is discussed further in the next section.

## Case Study Design

The case study was conducted in a network of four SCAs. They do business in the construction industry within the geographical area of four provinces in the middle of the Netherlands, an area covering half of the Dutch population. The study, running from June 2019 to November 2020, considers the circular use of timber and timber components in construction projects of these SCAs. In the Netherlands, timber is an increasingly promoted and used material in such projects as a response to the need to reduce CO_2_ and NO_x_ emissions [25, 26]. However, many uncertainties influence decision-making on how to materialize this ambition in practice. For example, the precise ecological gaining as compared to the use of other materials, the (in)stability of the (inter)national supply of dismantled timber, the uncertain quality of dismantled timber and the costs of preparing for reuse, or the (changing) preferences of clients. Involved SCAs struggle with questions related to these and related uncertainties [24].

Two aspects are relevant for the design of the case study: (i) choices regarding the nature of the case study and (ii) requirements regarding the facilitation. Concerning the first aspect, Yin [27] argues that choices need to be made regarding case selection and design, the protocols for collection and analysis of data, and the validation process. Concerning the second aspect, Franco and Montibeller [28] mention the need to stimulate interactivity and participation by the actors, to create a safe environment that allows actors to freely debate their views and positions, to use visualisation techniques, and to make progress with a responsive attitude to the dynamics of the process. Choices regarding the case study and the requirements for facilitation are discussed next.

### Case Selection and Design

**Fig. 4:**
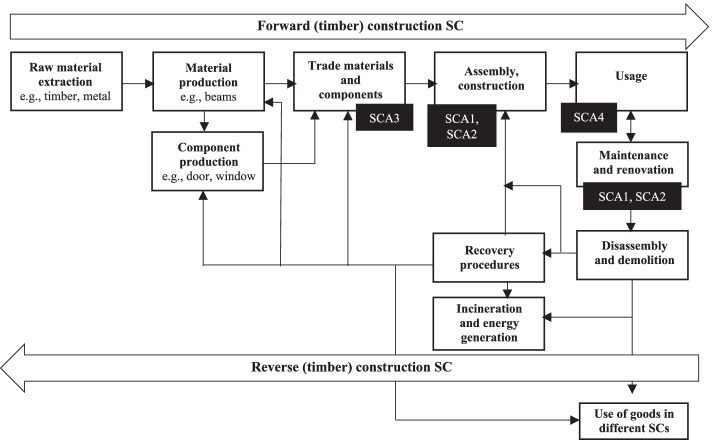
Positions of the participating SCAs in the Dutch construction supply chains

The case study follows an embedded design implying that the four participating SCAs are treated as multiple units of analysis, allowing for a detailed and comparative level of inquiry [27]. Three out of four SCAs are small- and medium-sized enterprises with various positions in the construction supply chain (Fig. 4). One is a housing organisation that often functions as client of the three SCAs. SCA1 focuses on technical maintenance of social housing estates. SCA2 concentrates on construction, maintenance, restoration, and interior projects in both the private and public sectors. SCA3 focuses on the trade of construction materials and components for private and public sectors. SCA4 is a housing corporation that builds, rents, and maintains around 14,000 units of (social) housing. On behalf of these four organizations, major decision-makers (general managers and owners) actively participated in the study.

The four SCAs were chosen for two reasons. First, the SCAs at present regularly join forces to construct, maintain, and renovate (social) housing estates and other buildings. Hence, they developed a basic level of trust in each other which is important for developing a shared strategy for implementing CLSC management. Second, at the start of the study, the actors had different attitudes towards CLSC management, which potentially allows for the collection of empirically rich data for studying the effects of the capability maturity approach. To clarify the second reason, initial interviews with the SCAs revealed that in the beginning of the study, two of the four SCAs appeared sceptical towards CLSC management questioning its feasibility, and none of the SCAs had yet set CLSC goals. This starting situation can, for instance, be illustrated by SCA1 stating: ‘I am sceptical towards CLSCs. It is not that I am against it, yet is it necessary? If so, I have no idea how and where to start’. Whereas SCA2 argued ‘we are intrinsically motivated to start a more circular business, although it is still only a business agenda item within our organisation. Goals are not formulated yet’. The discussion with the SCAs on the structure and aim of the case study resulted in the joint decision to consider the specification of CLSC goals for these SCAs being the main aim of the study. Given their starting positions, the SCAs agreed on the need to start exploring the uncertainties related to the possible responses of their supply chain to emerging grand challenges. This exploration was a first step towards becoming more aware of the need for change towards CLSC management (Maturity Stage 1, Fig. 2). Exploring these uncertainties should support the SCAs in taking the next step, i.e. specifying feasible CLSC goals for themselves and their supply chain network (Maturity Stage 2, Fig. 2). It was also explained to the actors how they would be supported methodologically and what kind of underlying data analyses could be performed in this initiating phase of the case study. Furthermore, it was explained that the first author would function as the main researcher and facilitator of the study. The SCAs’ agreement on the case study aims and the planned process and analytical steps towards that aim implied a limitation in the scope of the case study to the first two stages of the maturity development model. In terms of the baseline maturity assessment, the starting positions of the SCAs imply that they all started at Maturity Level 1 with respect to the management activities of Maturity Stages 1 and 2.

### Gathering Data

The data collection strategy applied in this study was based on a theory-based protocol with predefined questions and measurement scales [27]. Hence, the collection of data was based on the questions and management activities (Table 1) and a set of varying methods (Table 2) elaborated in [10] for the Maturity Stages 1 and 2 of the CLICK methodology. Table 2 also presents the timeline.

**Table 1:** Part of the conceptual framework of the CLICK methodology, adapted from Coenen et al. [10]

CLICK methodology				
*Maturity stages*	*Capabilities*	*Management activities*	*Levels of maturity*	*Analytical and evaluation questions*
1 Understanding and accepting responses to possible futures	Gained awareness of possible complex business-as-usual (BAU) SC behaviours over time, at geographical scales and/or organizational levels, in response to relevant possible futures	1a Identification of plausible futures, accepting the (relatively) most important futures for further study	1 Ignorance about the plausible futures	1. **Analytical**: Which grand challenges could affect a transition towards a CLSC for the SCAs involved?
			2 Gained awareness on and being able to identify plausible futures	2. **Analytical**: What are the causes of the challenges?
			3 Agree or agree to disagree on those futures that are perceived as important for further study	3. **Analytical**: What are the links between the driving forces of a single grand challenge and those among the challenges?
				4. **Analytical**: How can grand challenges unfold over time, at geographical scales and/or organizational levels?
				5. **Evaluation**: What are the (relatively) most important/relevant plausible futures for the SCAs involved for further study?
		1b Identification of the causal structures (i.e. causal interactions and feedback loops) of and multiple dimensions in linked grand challenges-BAU SC system models	1 Ignorance about the causal structures and multiple dimensions in relation to the models	6. **Analytical**: What are the boundaries of the BAU SC system model in terms of geographical scale(s), time horizon, and organizational level(s)?
			2 Gained awareness on and being able to casual structures in relation to the models	7. **Analytical**: What are the activities, endogenous, and exogenous variables of the BAU SC system model?
				8. **Analytical**: What are the causal interactions and feedback loops among the endogenous and exogenous variables of the BAU supply chain system model?
		1c Recognition of deep uncertainty of diverse types of complex SC models behaviours in response to the possible futures	1 Ignorance about the uncertainty of diverse types of complex SC model behaviours	9. **Analytical**: What are the ranges of uncertain parameters in relation to the main causal structures?
			2 Gained awareness on and being able to recognize the uncertainty of diverse types of complex SC model behaviours	10. **Analytical**: How can the BAU SC system models economically, ecologically, and/or societally behave over time at geographical scales and/or organizational levels in response to the possible futures?
				11. **Analytical**: Which combinations of deep uncertainties cause the diverse types of dynamic BAU SC behaviours?
2 Clarifying ambition to change	Gained awareness of, develop, and be able to accept the CLSC goals under deep uncertainty	2a Identifying possible/plausible threats and opportunities and considering multiple dimensions and accepting the (relatively) most important plausible threats and opportunities in the BAU SC system models	1 Ignorance about the possible/plausible threats and opportunities	12. **Analytical**: Which possible/plausible threats and opportunities in the supply chain can occur over time, at different geographical scales and/or organizational levels, from the economic, ecological, and/or societal perspective?
			2 Gained awareness on and being able to identify possible/plausible threats and opportunities and considering multiple dimensions	13. **Evaluation**: What are the (relatively) most important possible/plausible threats and opportunities for the SCAs involved to include for CLSC goal setting?
			3 Agree or agree to disagree on the main possible/plausible threats and opportunities	
		2b Identifying CLSC goals and accepting the (relatively) most important CLSC goals	1 Ignorance about CLSC goal setting	14. **Analytical**: Which economic, ecological, and/or societal goals need to be formulated to tackle the most important threats and opportunities in the SC over time at different geographical scales and/or organizational levels?
			2 Gained awareness on and being able to set CLSC goals, i.e. in addition to economic goals, also ecological and/or societal goals have been set, and at least two of the three other dimensions (e.g. time horizon) are considered	15. **Evaluation**: What are (relatively) most important CLSC goals for the SCAs involved?
			3 Agree or agree to disagree upon the main CLSC goals

**Table 2:** Overview of methods for the case study

CLICK methodology		
*Questions asked to gather data (Table **1****)***	*Methods/assignments*	*Schedule*
Questions 1 to 4 and 6 to 8	Individual interviews. The interviews were transcribed	June 2019
Questions 1 to 8	Group discussion. Discussion was transcribed	July 2019
	Collaborative causal modelling (workshop). The workshop was transcribed	September 2019
Question 5	(i) Collective development of plausible future scenarios (little versus much change); (ii) individual assessment of importance of plausible futures, (iii) group meeting to discuss results/disagreements, discussion was transcribed; and (iv) decision-making on the main plausible futures via email	October 2019
Question 9	(i) Desk research and (ii) meeting with professor in the field of forestry and an expert in processing dismantled timber	October to November 2019
Questions 10 and 11	(i) Exploratory system dynamics modelling and analysis performed by analyst (ESDMA) and (ii) individual presentations given by analyst to SCAs. Presentations were transcribed	December 2019 to April 2020
Question 12	(i) Individual identification of (uncertain) opportunities (O) and threats (T) for developing a CLSC and (ii) group meetings to discuss results. Group discussions were transcribed	May 2020
Question 13	(i) Individual identification of strengths and weaknesses of own firm and sector regarding the O and T, (ii) confrontation matrix based on SWOT analysis, (iii) individual assessment of strategies for handling O and T based on which the main O and T could be identified by the analyst, (iv) individual assessment of importance of proposed main O and T, and (v) group meeting to discuss the results/disagreements. Discussion was transcribed	June 2020
Question 14	(i) Group meetings and (ii) goal tree analysis. Meetings were transcribed	July 2020
Question 15	(i) Individual assessment of importance of CLSC goals and (ii) group meeting to discuss the results/disagreements. Meeting was transcribed	September to October 2020

### Post-Case Evaluation

The facilitated case study allowed to investigate the process of learning and the development of maturity following the afore-described process and measurement. As argued in the “Theoretical Background of the Systematic Capability Maturity Approach” section, this refers to situational maturity. To explore whether this situational maturity also contributes to the development of mental maturity, the SCAs were individually interviewed using a semi-structured interview guide with both open and closed evaluative questions directly after finalizing the case study (Table 3). In these post-case interviews, the added value of the applied analytical and collective methods was also discussed with the SCAs.

**Table 3:** Semi-structured post-evaluative interview guide

	Questions post-case evaluative interview	Reflecting on management activities
1	Have the causal loop diagrams (CLDs) helped you to understand various causal interactions and feedback loops in and between the different grand challenges and the (timber) construction supply chain? If not, why not?	1a/b
2	Since the group discussion of the CLDs, have you discovered any new causal interactions and feedback loops between grand challenges and CLSC management in construction? If so, what have you done with those identified causal interactions and/or feedback loops?	
3	During that group discussion, we paid attention to the influence of the dimensions ‘time horizon’, ‘geographic scale’, and ‘organizational level’ on the different variables in the CLDs. Do you think that since that group meeting you pay more attention to those dimensions when you think of a grand challenge and the possible consequences on CLSC management in construction? If so, can you explain this further? If not, why not?	
4	Have the graphics of SC model behaviours helped you to understand the uncertainty in relation to the different outcomes? If not, how do you perceive these outcomes (fixed, (un)likely)?	1c
5	Have the graphics on SC model behaviours helped you to formulate opportunities and threats? If not, why not?	2a
6	During the group meeting in which we discussed the opportunities and threats, we paid attention to the dimension’s ‘economy’, ‘ecology’, ‘society’, ‘time’, ‘geographic scale’, and ‘organizational level’ in relation to those opportunities and threats. Do you think that since that group meeting you pay more attention to those dimensions when you see opportunities and threats in relation to CLSC management in construction? If so, can you explain this further? If not, why not?	
7	Did the goal tree analysis help you to identify CLSC goals in which you also explicitly take multiple dimensions into account, or did you not need the goal tree for this? If so, can you explain this further? If not, why not?	2b
8	Various futures, opportunities, and threats, as well as CLSC goals were considered important by one SCA for further steps, while they were less important for the other SCA. To arrive at a compromise (at least to try), a group discussion was organized. What do you think of this method?	1a; 2a; 2b
9	Do the chosen CLSC goals motivate you to develop CLSC interventions? If not, why not?	General

### Data Analysis

Three steps were taken to analyse the data. *First*, the analyst used an a priori established coding book [29] (Table 4) to assess maturity levels achieved by the individual SCAs and establish how deep uncertainty and dynamic complexity affected the decision-making processes of the individual actors. The coding was based on insights from literature on decision-making processes [e.g. 30] adopted in the classification of the different maturity levels of the CLICK methodology. This literature underlines that there are moments of ‘recognition’ and ‘identification’ (linked to maturity at Level 2), followed by moments of ‘choice/making decisions’ (linked to maturity at Level 3). The notions of recognition and identification refer to two distinct aspects. Firstly, they refer to whether statements by the SCAs can be interpreted as deterministic, probabilistic, or as possible/plausible. In the case of determinism, SCAs would use words such as ‘for sure’ or ‘definitely’ or ‘certainly’. In the case of probability, words like ‘probably’, ‘likely’, or ‘unlikely’ are used. In the case of possibility/plausibility, SCAs use words like ‘reasonable’, ‘plausible’, ‘possible’, or ‘credible’. Secondly, they refer to whether statements by the SCAs include mentioning dynamically complex issues (such as causal interactions and feedback loops in supply chains) and multidimensionality (e.g. in relation to CLSC goals). Next, the notion of choice/decision-making refers to taking decisions and making choices by the SCAs on how to continue in the process. These decisions can be classified in terms of ‘agreement’, ‘disagreement’, or ‘agree to disagree’ [14, 31], ‘wait-and-see’, and ‘attack’ or ‘defend’ [24, 32].

**Table 4:** Operationalisation of constructs of observation

Management activities	Constructs	Measurable variables (i.e. codes/values)
1a/ 1b	Identification/recognition	Grand challenges (yes or no), causal interactions (yes or no), feedback loops (yes or no), multiple dimensions in relation to causal structures (yes or no)
	Choice/decision-making	(Relatively) most important future scenarios relevant for further research (agree, agree to disagree, or disagree)
1c	Identification/recognition	Uncertainty of supply chain model behaviours (likely/unlikely, certain/fixed, or possible)
2a	Identification/recognition	Opportunities and threats (likely/ unlikely, certain/ fixed, or possible/plausible), multiple dimensions in relation to opportunities and threats (yes or no)
	Choice/decision-making	(Relatively) most important opportunities and threats (agree, agree to disagree, or disagree)
2b	Identification/recognition	Multiple dimensions in relation to CLSC goals (yes or no)
	Choice/decision-making	(Relatively) most important CLSC goals (agree, agree to disagree, or disagree)
1a, b, c; 2a, b	Choice/decision-making	Decision-making in-the-moment in the research process (wait-and-see, attack/defend, and/or knowledge acquisition)

The *second* analytical step involved cross-unit comparison, with the aim to identify patterns of similarities among, and differences between, the different SCAs. Differences in maturity growth between the more sceptical SCAs and the more motivated SCAs emerged. Identifying and reflecting on these emerging patterns enables statements about the added value of the systematic capability maturity approach for attaining situational maturity. The *third* analytical step involved a comparison between the findings of the post-case evaluative interviews and the observations made during the case study. The aim of this step was to explore whether situational maturity within the case, also contributed to the development of mental maturity, hence influencing business beyond the case.

### Validation

By presenting empirical findings in the “Findings” section, following the predefined steps, this study tries to keep the ‘chain of evidence’ and the link between the findings and conclusions as transparent as possible. Furthermore, a case study database was put together, consisting of raw data documents and the a priori code book document, enabling retrieval for future investigators [33]. Finally, the (facilitated) case study protocol mentioned above, based on the elaborated framework, contributes to the reliability and replicability of the study.

### Facilitation

With this predefined data collection strategy, the researcher acted as an analyst in what Franco and Montibeller [28] label as the ‘modelling space’. This activity produced outcomes such as system responses and priorities for action. The researcher also functioned as a facilitator in what these authors label as the ‘group process space’, producing outcomes such as commitment to action or learnings. Four facilitation principles were applied [28]. *First*, the researcher should stimulate interactivity and active participation by the different actors because the process of debating arguments and positions is considered key to learning. To that end, the case study followed a mixed strategy of individual assignments and group sessions. Individually performing an assignment stimulates the SCAs to elaborate their view on the issue at hand. This was followed by discussing the different views in a group meeting, allowing the actors to either get confirmation for their view or adopt arguments to change their views and positions. *Second*, a safe environment for the participants should be created, since they should feel free to share and change their ideas without being defensive. In the case study, a safe environment was created by organizing the meetings in a university’s conference room, by providing data and model results in response to the process dynamics, and by not interfering in the arguments used in the debate among the SCAs. The basic trust of the SCAs in each other also contributed to the creation of a safe environment. *Third*, various visualization techniques should be used to deal with complex information. In the case study, tools such as causal loop diagrams, decision graphs, and value trees were extensively applied. *Fourth*, the researcher should take care for making process: get the intended steps made and tasks performed without losing responsiveness to the dynamics. This requires awareness of tensions and conflicts and applying means to clarify and solve these. Therefore, in this study, debates were enriched with data, evaluative moments were introduced, and after each step taken perspective was given on the next steps to be made.

## Findings

Table 1 provides the structure of the process followed in the case study: analyses first focus on Maturity Stage 1 (exploring plausible futures and their impacts) followed by Maturity Stage 2 (specifying goals). These analyses are respectively described in the “Maturity Stage 1” and “Maturity Stage 2” subsections. The management activities in these subsections refer to column 3 in Table 1, whereas the maturity levels are described in column 4 of that table. Finally, in the “Post-Case Evaluation” section, the findings of the post-case interviews are presented.

### Maturity Stage 1

#### Management Activities 1a and 1b, Maturity Levels 1 and 2

The learning process pursued by the SCAs is related to the identification of plausible future states of the world in this first step of the case study. This involves the identification of four core elements by the SCAs: grand challenges, causal interactions, feedback loops, and multiple dimensions in the link between grand challenges for a CLSC and the business-as-usual (BAU) supply chain system. To which extent the SCAs were able to identify g*rand challenges* and causes of these challenges was investigated in individual interviews. All SCAs appeared aware of the grand challenges influencing their supply chain. They mentioned ‘Dutch climate agreement’, ‘Dutch resource agreement’, ‘geopolitical stability’, ‘CLSC technological innovation in the Dutch timber industry’, ‘global availability of timber’, ‘tight labour market in the construction supply chain’, and ‘global climate change’.

**Fig. 5:**
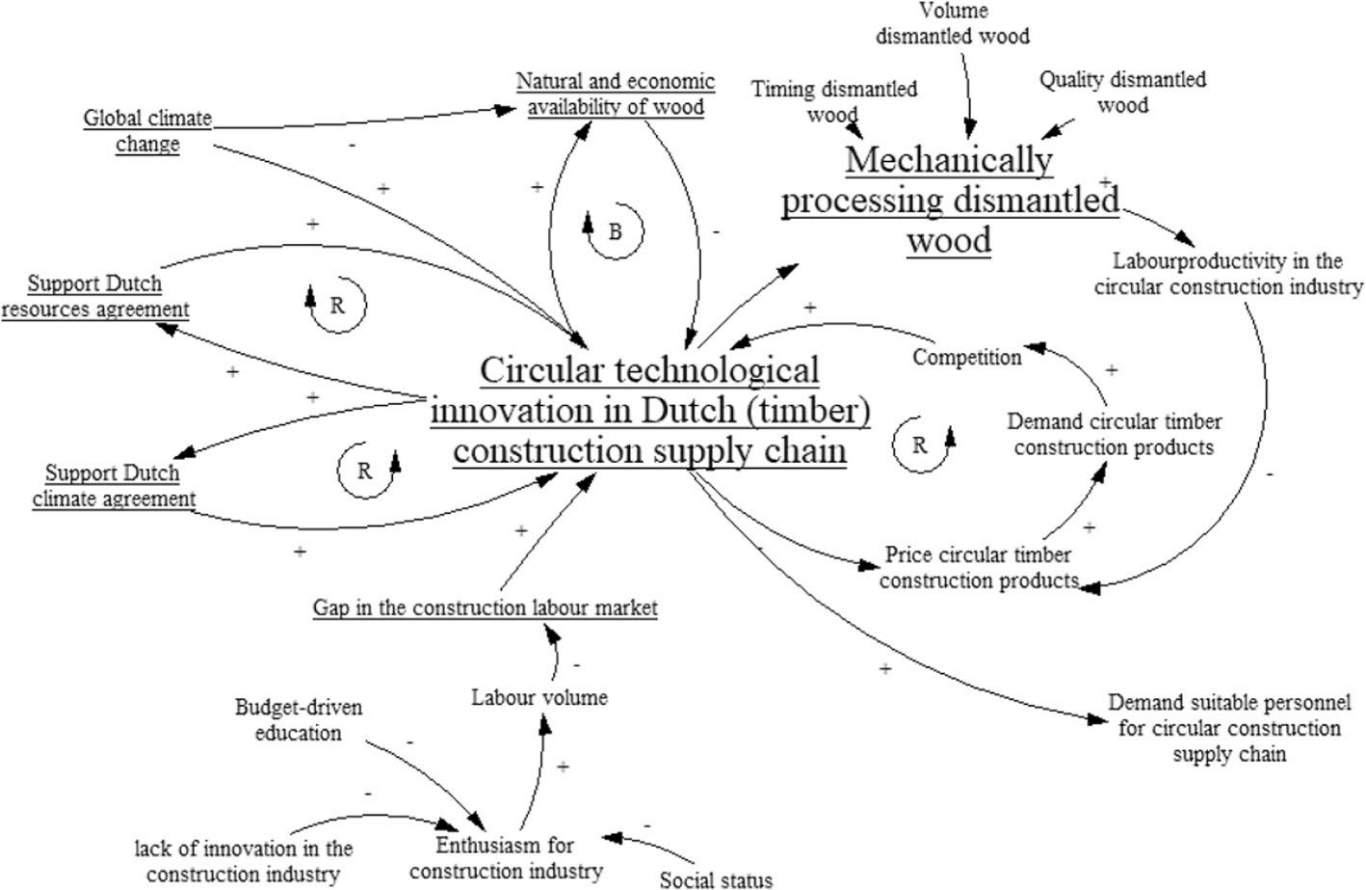
Example of one of the causal loop diagrams: technological innovation in the CLSC

Based on this information and input from the literature, in the next step, the researcher constructed basic causal loop diagrams (CLDs) of each of the afore-mentioned grand challenges in relation to the construction supply chain. This was done to trigger debate on the possible impact of the challenges on the dynamics in their supply chain. CLDs aim to visualize the causal structures in a system in terms of *causal interactions and feedback loops* [34]. For example, Fig. 5 shows the CLD of the challenge ‘CLSC technological innovation’, specifically in relation to mechanically processing dismantled timber. The main grand challenge (larger font size) and other connected challenges are underlined. Other variables represent the driving forces behind the main grand challenge and the possible effects on the timber construction supply chain. A causal effect from one variable to another is positive ( +) when a change in one variable leads to a change in the other variable in the same direction. Conversely, a causal effect from one variable to another variable is negative ( −) when a change in one variable leads to a change in the other variable in the opposite direction. The ‘R’ refers to a reinforcing loop, generating system behaviours such as growth and collapse at an ever-increasing rate. The ‘B’ refers to a balancing loop that generates stability [34].

The basic models were discussed in a group meeting. From the perspective of learning, the analysis focused on the degree to which SCAs were able to grasp the dynamics of the system and to enrich the causal model. All SCAs were able to identify additional causal interactions in and between different CLDs. For instance, SCA2 argued that there is interaction between resource availability and geopolitical stability: ‘What I miss [in the CLD related to global availability of timber], but that has to do with geopolitics, is that the US has imposed import duties on Canadian timber since 2017. If this continues, the demand for European timber from the US could increase, resulting in a rising timber price in Europe’. SCA1 and SCA4 also identified additional feedback loops. For example, regarding the CLD of the grand challenge ‘global climate change’, SCA1 stated ‘I think there are now reinforcing elements that make the current climate change serious. The increasing amount of forest fires increases the amount of CO_2_ emissions globally, and that accelerates climate change. I do not see that R-loop in the CLD. We should add it’. The group discussion also shows that both SCA2 and SCA3 were able to simultaneously consider *multiple dimensions*. SCA2 mentioned the geographical scale and organizational level, while SCA3 considered the time horizon and geographical scale. SCA1 and SCA4 only addressed the time horizon in their views.

Since all SCAs were able to identify three out of the four abovementioned core elements of plausible futures, it can be concluded that, in this phase of the process, the SCAs showed growth from Maturity Level 1 to Maturity Level 2.

#### Management Activities 1a and 1b, Maturity Level 3

Learning, in this step in the case process, dealt with exploring the relative importance of the potential impacts of plausible future on the SCAs’ supply chain and based on that selecting those futures that should be explored in-depth. The SCAs first individually rated the importance of the plausible futures. Next, a group meeting was organized to discuss the main differences between the individual ratings and to try to reach a collective agreement on the futures that would be made subject of further study.

The SCAs notably disagreed on two plausible future states of the world, described in Table 5. SCA3 rated both states as being not very important, while the other SCAs rated them as very/extremely important. Regarding plausible future state A, SCA3 questioned whether the timber industry and related transportation emits so much CO_2_ that it would lead to higher prices if CO_2_ taxes would keep rising. However, the debate with the other SCAs caused changes in SCA3’s view, resulting in support for the inclusion of A in the set for further study. As for plausible future state B, SCA3 argued ‘I do not believe that technologies will be developed for dismantling and cleaning timber. At least not if the quality and quantity of this timber remains so uncertain. However, you can include it because it may indeed be the case that, as SCA2 mentions, technology is key to successful CLSC management’.

**Table 5:** Plausible future states of the world that generated disagreement

	Plausible future states of the world			Answersgivenbyactors involved			
		*Minor change*	*Major change*	*SCA1*	*SCA2*	*SCA3*	*SCA4*
CLSC technological innovation	A	In the next 25 years, the development of CO_2_ reducing techniques in the timber industry and related transport sector remains limited. As a result, the reduction of CO_2_ emissions in those sectors is also limited. This has a negative effect on the amount of CO_2_ taxes that must be paid by those sectors, and the import price of coniferous sawn timber and plywood and non-coniferous plywood	CO_2_ reducing techniques will be developed in the coming 25 years, which will reduce CO_2_ emissions in the Dutch timber industry and the related transport sector. This has a positive effect on the amount of CO_2_ taxes that must be paid by those sectors, and the import price of coniferous sawn timber and plywood and non-coniferous plywood	VI	VI	NVI	VI
	B	In the next 25 years, dismantled timber and sheet material will be processed by hand, keeping labour productivity low. As a result, the price of dismantled timber and sheet material remains high, only a limited amount of CO_2_ will be stored longer, and incineration of that material remains high and will make extraordinarily little contribution to the demand of the (semi) public sector for closed-loop timber roofs and facades	In the Netherlands, significantly more dismantled timber and sheet material will be processed mechanically over the next 25 years, increasing labour productivity. This lowers the price of dismantled timber/ sheet material, increases the longer storage of CO_2_, decreases incineration of that material, and contributes to the demand of the (semi) public sector for closed-loop timber roofs and facades	EI	EI	NVI	EI

*VI* very important, *NVI* not very important, *EI* extremely important.

The result of this meeting was that the SCAs agreed upon the set of plausible futures that are collectively perceived as important for their CLSC management strategy and therefore should be made subject of further study. This observation supports the conclusion that after the analytical steps made in the case process, the SCAs’ maturity in dealing with Activities 1a and 1b were at Maturity Level 3.

#### Management Activity 1c, Maturity Levels 1 and 2

The case study continued with an in-depth analysis of supply chain model behaviour under different conditions of the afore-selected most relevant plausible futures. To that end, the researcher developed three economic and two ecological quantitative simulation models as a basis for observing learning effects when confronting SCAs with the results of the simulations. The analysis of the learning process focused on how the SCAs interpret the modelled future supply chain behaviours. Are the SCAs able to keep considering the futures and their impact as possible, recognizing deep uncertainty regarding probability of occurrence?

**Fig. 6:**
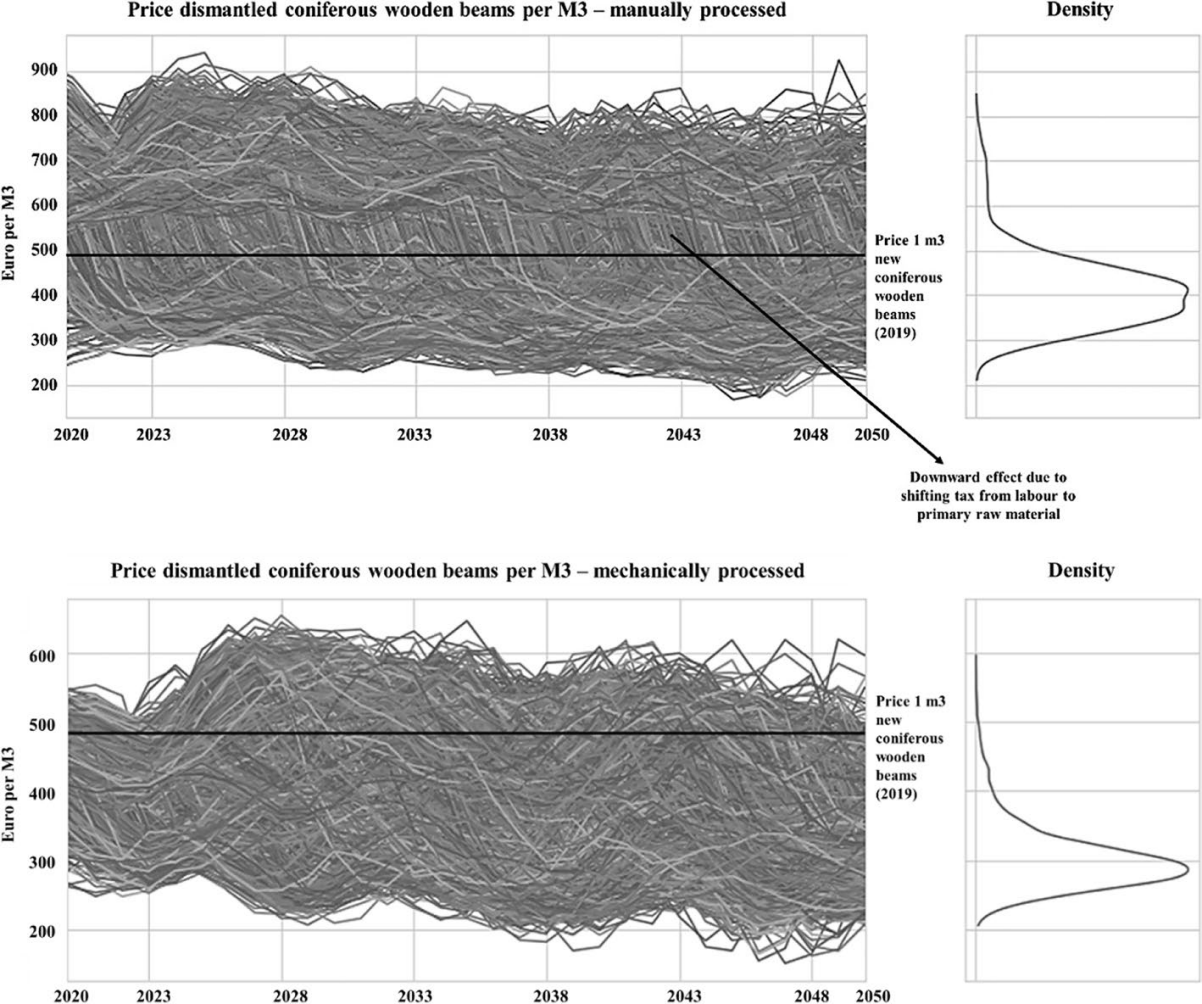
Dynamic behaviour of price per m.^3^ of processed dismantled coniferous timber beams

The simulation models consist of stock and flow diagrams and variables that are based on the CLDs that were constructed in interaction with the SCAs and turned into equation-based models. These models consist of many distinct functions (e.g. polynomial, Gaussian and S-shaped curves, time delays) to represent the expected (according to relevant literature) relationships between the included variables, consequently influencing the behaviour of the models. To perform the simulations, exploratory system dynamics modelling and analysis (ESDMA) was used [35], enabling the generation of thousands of scenarios based on the structural features of plausible futures. Each simulation model includes a set of deeply uncertain parameters. For example, Table 6 gives an overview of various major uncertainties in parameters and structure related to one of the economic simulation models. This model explores, among other things, how changes in the (i) volume, quality, and timing of dismantled coniferous timber beams and (ii) Dutch tax system in terms of shifting from tax on labour to tax on primary raw materials might affect the price of 1 m^3^ manually and mechanically processed dismantled coniferous timber beams in the upcoming 32 years (2018–2050). The results of this specific model are presented in Fig. 6, following common presentation practices in ESDMA. They were, together with the results for the other models, presented to each of the SCAs in bilateral meetings. Hence, Fig. 6 is just one result out of a set of similar types of figures.

**Table 6:** Various uncertainties related to one of the three economic simulation models

Name	Range and model structure
Mechanically processed metres of dismantled coniferous timber beams per hour	200–300 m
Manually processed metres of dismantled coniferous timber beams per hour	25–35 m
Varying quality, quantity, and timing mechanically processed dismantled coniferous timber beams	Polynomial model structure with uncertain parameters (e.g. 5 to 99% of timber approved)
Varying quality, quantity, and timing manually processed dismantled coniferous timber beams	
Shifting payroll tax rate	75 to 95%
Timing payroll tax rate	10 to 22 years

Based on the presentation of the simulation results, only SCA4 interpreted the results as ‘possible’ (hence deeply uncertain), whereas the other SCAs gave a probabilistic interpretation. For instance, SCA3 argued ‘I think that the price of dismantled timber will remain higher than the price of new timber, because the quality and volume of that timber is just not meeting the standards. The few scenarios that show a lower price are unlikely to happen in my opinion’. SCA2 seemed to already have clear expectations and to stick to these: ‘I am not surprised about these results. CLSC becomes increasingly important. Timber prices will increase in the future, especially if climate change keeps on accelerating. That is a fact. I do not think that the price remains the same or close to what it is now, because the changing climate will create scarcity’. Hence, most SCAs found it difficult to interpret the simulation results in terms of ‘possibility’ which is the basic purpose of ESDMA. To them, the density graphs suggested that certain system behaviours are more likely to occur than others. This observation may indicate a discrepancy between the purpose of ESDMA analyses and the way decision-makers are inclined to interpret the resulting information. It could also indicate a cognitive difficulty of SCAs to clearly distinguish between the concepts of possibility (being core in the deep uncertainty discourse) and probability (strongly related to the traditional way of thinking). Further in the process, when thinking about the opportunities and threats (see “Maturity Stage 2” section), it was observed that SCA1 and SCA3 after all inclined to interpret the dynamic behaviours as ‘possible/uncertain’, suggesting that the SCAs need at least time to internalize the difference of both concepts.

Based on observations of the SCAs’ decision behaviour in relation to Management Activity 1c, it was concluded that SCA1, SCA3, and SCA4 have grown from Maturity Level 1 to Maturity Level 2, whereas SCA2 remained at Maturity Level 1.

#### Capability Achieved

**Fig. 7:**
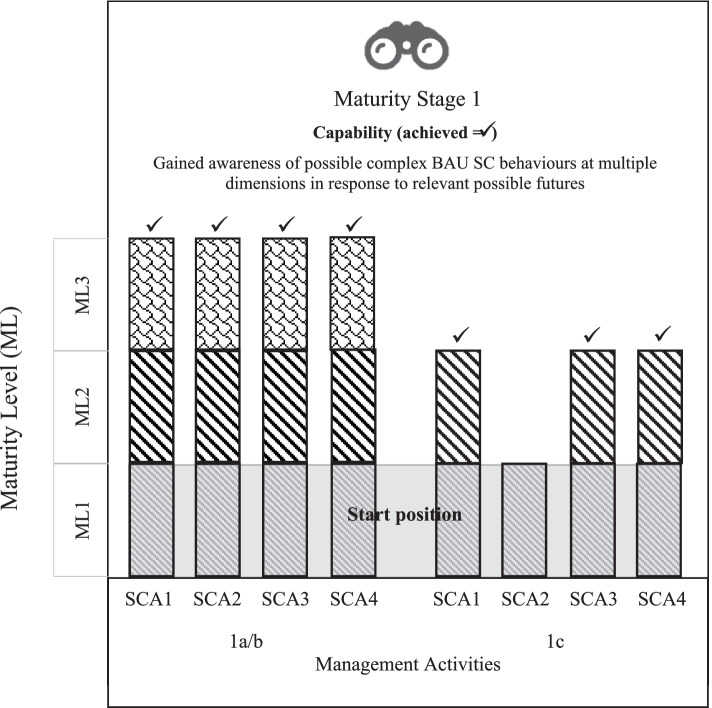
Maturity Stage 1: maturity levels achieved by the SCAs

Based on the findings regarding Management Activities 1a, 1b, and 1c, it can be concluded that all four SCAs went through a process of learning on complexity and uncertainty. The observations on their (changing) views and arguments used can, according to the predefined evaluation protocol, be interpreted as a growth in maturity in dealing with the activities related to Maturity Stage 1. Only SCA2 did not fully achieve the predefined capability related to Maturity Stage 1. The actor had considerable difficulties with recognizing the deep uncertainties surrounding the supply chain system behaviours. The conclusions on attained maturity levels are summarized in Fig. 7.

### Maturity Stage 2

Following the structure of the CLICK methodology, the next phase in the case study focused on the SCAs’ learning to specify goals for moving towards CLSC management in their own business as well as jointly in the supply chain. As shown in Table 1, this involved performing two activities.

#### Management Activity 2a, Maturity Levels 1 and 2

This activity required the SCAs, based on the in Stage 1 provided information and gained insights, to identify opportunities and threats for (their role in) their supply chain and to indicate how they interpret these in terms of uncertainty. The SCAs started with performing this activity individually. All SCAs appeared to be able to identify various (deeply uncertain) opportunities and threats (see Table 7), mentioning that they have used the ESDMA results, information in earlier debates and other sources such as newspapers/media, and practical experiences. It was observed that SCA1, SCA3, and SCA4 interpreted various deeply uncertain opportunities and threats correctly as ‘plausible/ possible’, whereas SCA2 interpreted most of them as ‘deterministic’.

**Table 7:** Main opportunities and threats

Main opportunities	
O1	Creating market share in the trade of reusable timber construction products and to a lesser extent in dismantled timber and dismantled timber sheet material
O2	Generating and exchanging knowledge and experiences about CLSCs (in relation to timber, timber roof constructions, and timber facades) to convince clients and the internal organization of the value of closed-loop construction supply chains. This conviction can then lead to circular assignments, which can increase turnover and save CO_2_
O3	Dynamics in relationships between actors: actors are challenged to take on distinct roles or are forced to do so in CLSCs
O4	Preparing for a possible new policy in the Netherlands or EU that promotes (longer) CO_2_ storage in timber
O5	Investing in a significant improvement of the trade in reusable timber construction products and to a lesser extent in dismantled timber and dismantled timber sheet material to increase turnover and market share in those products
O6	Creating a movement towards closed-loop construction supply chains
O7	Crises (e.g. COVID-19, PFAS, financial) as stimulus for closed-loop timber construction
Main threats	
T1	Increase in government taxes for housing corporations and political pressure on lower rents possibly limits attention and resources for closed-loop (timber) construction
T2	Continuing/deep uncertainty about the volume, timing, quality and price of solid dismantled timber, and sheet material
T3	Current negative imaging (closed-loop) timber construction
T4	Crises (e.g. COVID-19, PFAS, financial, national housing shortage) as a reason for postponing closed-loop timber construction. Grabbing for standard construction methods on the short term—and not innovating in the field of closed-loop (timber) construction—is lurking

These inputs were further processed by the researcher and discussed in a group meeting, which caused SCA2 to realize that opportunities and threats should indeed be considered ‘plausible/possible’. An illustration is for instance that SCA2 first claimed that quality and volume of dismantled timber are insufficient in practice and therefore forms a definitive threat when it comes to taking steps towards CLSCs: ‘There is not enough usable timber available for reuse. It will take too much effort. The desired quality is too low’. However, the ESDMA clearly showed that the volume and quality of dismantled timber is deeply uncertain: while in a year sufficient dismantled timber may be available that meets the Dutch construction standards, in another year that may not be the case. Discussing this in the group, SCA2 recognized that indeed ‘insufficiency’ in relation to volume and quality is not a fact but an uncertainty, and for that matter, a strategy based on adaptive procurement of dismantled timber and buffering could also been seen as an opportunity.

Following the data collection protocol, observations were also made regarding the degree of multidimensional thinking, since acknowledgement of the ecological and/or societal dimensions in addition to the economic dimension is important for CLSC management [e.g. 36]. SCA3 and SCA4 appeared to be able to include both the time horizon and organizational level in the formulation of and discussion on the opportunities and threats. SCA1 considered only the time horizon, while SCA2 focused only on the organizational level. SCA3 and SCA4, next to the economic opportunities and threats, also considered societal and/or ecological opportunities and threats. The other SCAs considered only economic opportunities and threats.

The observations in relation to the SCAs’ performance of Management Activity 2a led to the conclusion that SCA3 and SCA4 developed a maturity at Level 2, whereas SCA1 and SCA2 remained at Maturity Level 1 given their limited awareness of multidimensionality.

#### Management Activity 2a, Maturity Level 3

After the identification of opportunities and threats, the SCAs were asked to individually identify strengths and weaknesses of their own organisation in relation to the identified opportunities and threats. Thereafter, the opportunities and threats were confronted with the strengths and weaknesses, to challenge the SCAs to individually think about individual as well as collective strategies (e.g. wait-and-see, defend, attack) for dealing with the numerous opportunities and threats. Based on the individually generated information, the main opportunities, and threats for the SCAs could be identified (Table 7). The SCAs were individually asked if they agree with the resulting list. Disagreement among the SCAs about the (un)importance of five (combinations of) opportunities and threats was observed (see Table 7: O2, T2, O7 and T4, O1 and O5, O4). In a subsequent group meeting, three out of five disagreements were resolved by achieving an agreement based on the exchange of arguments; the discussions on the two other disagreements resulted in ‘agree to disagree’. Concerning the latter, for instance, SCA4 disagreed that crises such as the Dutch NO_x_ crisis, PFAS crisis, and COVID-19 are not a major threat for closed-loop timber construction supply chains: according to SCA4, these kinds of crises could lead to procrastination among actors. The other SCAs, however, argued that their choice to remain actively involved in this case study during the outburst of the COVID-19 crisis in 2019/2020 and unexpected restrictions on building due to NO_x_ emission problems was sufficient evidence that such crises would not cause procrastination. SCA4 still disagreed, resulting in the decision by all SCAs to add emerging crises to the list of stimuli of closed-loop timber construction and as a possible reason for procrastination.

The conclusion on basis of the observations from this step in the analysis is that in relation to Management Activity 2a, all SCAs achieved Maturity Level 3.

#### Management Activity 2b, Maturity Levels 1 and 2

The next step in the process involved exploring whether the SCAs were able to identify CLSC goals for their supply chain. To be able to make this step, the theoretical view is that the SCAs, based on the previous steps in the process, should have developed the ability to (i) set ecological and/or societal goals for CLSC management next to economic goals and (ii) simultaneously take the dimensions time horizon, geographical scale, and organizational level into account. To observe whether this was the case, first, a group meeting was organized to trigger the SCAs to start with the formulation of CLSC goals for their supply chain. This resulted in a set of initial goals. After the group meeting and on the basis of the recorded discussion, these goals were rank ordered by the researcher in terms of a goal tree [37]. A goal tree analysis orders a set of goals in terms of a hierarchy of main goals, subgoals, and sub-subgoals, where goals at a lower level contribute to higher level goals. Next, the constructed goal tree was sent to the individual SCAs with the request to consider whether (and which) additional CLSC goals should be added to the goal tree and what geographical scale, organizational level, and time horizon can be assigned to each goal. The reply from the SCAs is summarized in Table 8, notably showing that SCA3 and SCA4 developed a multidimensional perspective on CLSC goals. For instance, SCA3 argued that it is important already in the present supply chain to meet the goals formulated in the Dutch climate and resource agreement, implying 50% CO_2_ reduction in 2030 as compared to 1990 and 50% less consumption of raw materials. SCA4 mentioned a societal goal related to the development of healthy homes, as well as an ecological-economic goal: ‘implement at least 50% of the projects initiated by the SCAs by 2030 in an ecologically circular and affordable manner’. Although SCA1 and SCA2 also took the time horizon, geographical scale, and organizational level into account, they, compared to SCA3 and SCA4, only identified economic goals.

**Table 8:** Dimensions involved in CLSC goal setting

CLSC goals	SCA1		SCA2		SCA3		SCA4	
	*Group meeting*	*Individual*	*Group meeting*	*Individual*	*Group meeting*	*Individual*	*Group meeting*	*Individual*
Economic goals	✓	✓	✓	-	✓	✓	✓	✓
Ecological goals	-	-	-	-	✓	-	✓	✓
Societal goals	-	-	-	-	✓	✓	✓	✓
Time horizon	-	✓	-	✓	-	✓	✓	✓
Geographical scale	-	✓	-	✓	-	✓	-	✓
Organizational level	✓	✓	-	✓	-	✓	-	✓

These observations suggest that in relation to the goal setting activity, SCA3 and SCA4 grew from Maturity Level 1 to Maturity Level 2, whereas SCA1 and SCA2 remained at Maturity Level 1 due to their limitation in goal setting to merely economic goals. They did not show a learning process that met the predefined criterion for a higher maturity level.

#### Management Activity 2b, Maturity Level 3

**Fig. 8:**
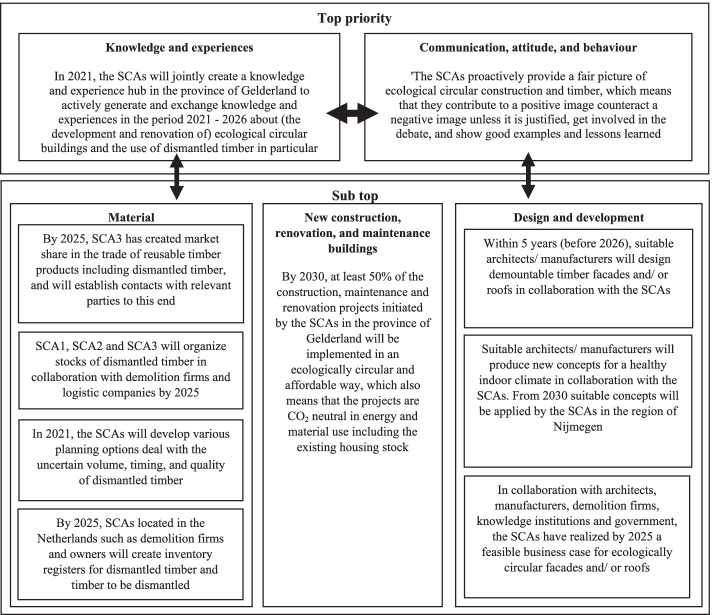
Major CLSC goals

The last step in the process involved the setting of priorities for acting by the SCAs. The analysis of learning in this context focused on the question whether the SCAs were willing and capable of making this step to enable observations in this respect, the SCAs were first asked to individually assess the importance of various goals in the CLSC goal tree for seizing the opportunities and resisting the threats. The assessments revealed disagreement among the SCAs on the importance of various CLSC goals. For example, one goal focused on designing demountable timber facades and/or roofs in collaboration with parties such as architects. SCA1 and SCA3 considered this a very important goal, while SCA2 and SCA4 did not agree. SCA2 and SCA4 argued that they were not skilled enough to design such facades or roofs. To resolve this and some other disagreements, a group discussion was organized where the SCAs were invited to exchange their views and arguments. By the end of the discussion all disagreements were resolved, and various goals had been modified, merged, or eliminated. Moreover, the goals were prioritized again. Two goals were given top priority in relation to ecological circular buildings and the use of dismantled timber (see Fig. 8): (i) the gaining and sharing of knowledge and experiences and (ii) changing the communication, attitude, and behaviour of SCAs.

The observations during this step in the case study showed that all SCAs experienced a learning process that helped them to attain a Maturity Level 3 for prioritizing CLSC goals.

#### Capability Achieved

**Fig. 9:**
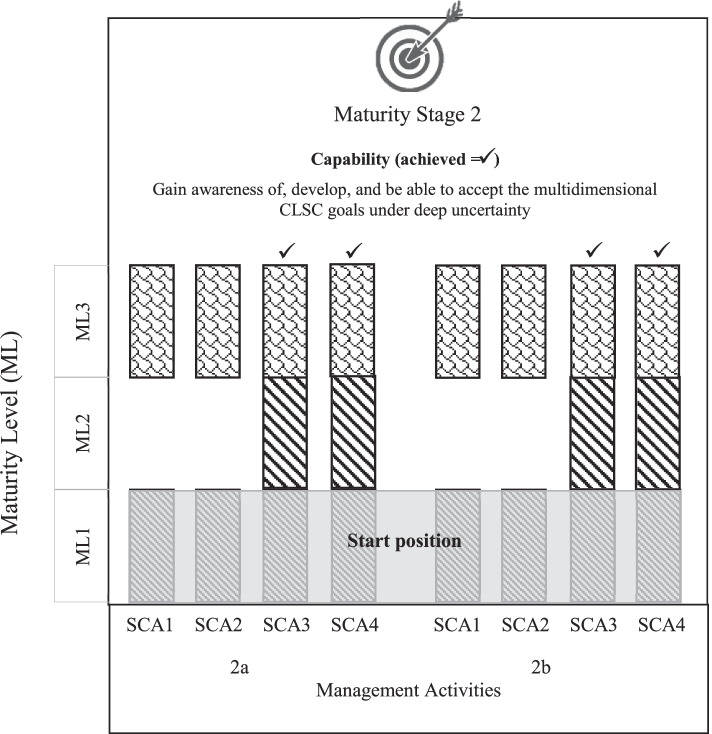
Maturity Stage 2: maturity levels achieved by the SCAs

Based on the observed performance of the Management Activities 2a and 2b, it was concluded that, different from SCA3 and SCA4, SCA1 and SCA2 did not fully achieve the capability that is theoretically related to Maturity Stage 2 (Fig. 9). These actors were unable to simultaneously include multiple dimensions when considering opportunities and threats as well as CLSC goals. Their focus remained primarily on the economic dimension. Nevertheless, SCA1 and SCA2 were in the end also able to agree upon the main opportunities and threats in a later phase of the process. Going through the process, they grew from Maturity Level 1 to 3 in this respect, skipping Maturity Level 2. This conclusion suggests that processes of learning and maturity growth are not necessarily as linear as assumed in theory.

### Post-Case Evaluation

As mentioned, (individual) post-case semi-structured evaluative interviews (see Table 3 for the design) were held shortly after closing the case, i.e. in November 2020. Aim was to observe to what degree situational learning effects in the case contributed to mental maturity and to evaluate to SCAs’ experiences with the followed process and use of different methods.

Regarding the issue of maturity, reflecting on Maturity Stage 1, SCA1 most explicitly stated to have become more structurally aware of the dimensions ‘time horizon’, ‘geographical scale’, and ‘organizational level’ after the various group discussions. However, all SCAs indicated to find it difficult to conceptualize such a multidimensional approach for their present practices. Reflecting on Maturity Stage 2, all SCAs stated that after closing the case, they still supported the agreed upon final sets of goals for moving towards CLSC management. Only SCA3 stated to keep doubts about the feasibility of the use of dismantled timber due to the many discussed uncertainties. Reflecting on the followed case study process, the SCAs mentioned that irrespective fast-changing circumstances (e.g. COVID-19, NO_x_ crisis) they experienced the steps made as supportive in their development of a sharper view on the feasibility of re-using timber and understanding of the many related uncertainties. Interestingly, all SCAs expressed their wish to continue the process in a joint follow-up study focusing on the next maturity stages, notably the selection of a set of no-regret interventions to achieve their main CLSC goals.

Regarding the applied methods, three observations are worth mentioning. *Firstly*, all SCAs reported to have experienced the method—of first an individual assessment and then a joint discussion—as supportive and valuable for developing their views and joint decision-making. It helped them to obtain a better understanding of the other SCAs’ points of view, allowing for a constructive discussion and shared decisions on how to move forward. *Secondly*, although some of the SCAs (notably SCA1) did not consider the ESDMA results to be of great value for understanding deep uncertainty, it could be observed that throughout the case process, the simulation has triggered learning effects for the SCAs after all. For instance, SCA4 mentioned the influence of ESDMA on formulating uncertain opportunities and threats. *Thirdly*, the goal tree was explicitly mentioned by the SCAs as being a supportive tool when it comes to addressing the dimensions ‘time horizon’, ‘geographical scale’, and ‘organizational level’. The goal tree forced the SCAs to consider these dimensions in a more systematic way as compared to the more open debate in the group meeting.

## Discussion

Overlooking the described facilitated analytical process, two questions arise: (i) which patterns of maturity growth, decision-making, and double-loop learning can be observed, and (ii) what was the effect of the improved insights in deep uncertainty and dynamic complexity regarding dismantled timber-oriented CLSC management in the SCAs’ construction projects? The first question is addressed by looking at the differences between initially ‘sceptical’ SCAs versus ‘motivated’ SCAs. This is followed by a reflection on the concepts of ‘situational maturity’ and ‘mental maturity’. After that, the second question is addressed.

### Sceptical SCAs Versus Motivated SCAs

No notable differences could be observed regarding how deep uncertainty and dynamic complexity affected the initially sceptical SCAs and the motivated SCAs in terms of their decision-making or learning during the process. In line with other studies [11, 13], in this case too, the involved SCAs struggled with deeply uncertain issues, in particular when it came to uncertainties surrounding the quantity, quality, and the price of dismantled timber. That may explain why in the end knowledge related CLSC goals have received the highest priority. More knowledge is perceived by the SCAs as an effective way to deal with emerging ‘known unknowns’. As the sceptical SCA3 stated: ‘In the knowledge hub we may develop and test concrete solutions for the uncertainties surrounding dismantled timber. Now those uncertainties are too large to do anything with this timber’. Notwithstanding this hesitative approach, the attitudes of both the sceptical and the motivated SCAs regarding dismantled timber seemed to have changed during the process. While the sceptical SCAs remained reserved, they became more open and motivated to take steps towards CLSC management. In contrast, the initially strongly motivated SCA2 and SCA4, remained motivated, but became more aware of the uncertainties and complexities in relation to CLSC management for dismantled timber. For them, this resulted in some downscaling of ambitions and a more realistic attitude towards CLSC management of dismantled timber. This finding matches the expectation underlying the developed capability maturity approach that following a structured explorative process contributes to converging views and decisions of the involved SCAs.

Overall, the improved content-oriented insights tended to strengthen a ‘wait-and-see’ attitude among the SCAs towards changing the core business. For example, SCA1 stated that ‘the national government is fickle. They must take more responsibility and make circular procurement mandatory’. Furthermore, SCA4 argued that today other themes than circularity are more urgent within SCA4’s organization and that consequently external incentives (e.g. from public policies) are needed to get circularity higher on the organization’s agenda. This wait-and-see attitude does not yet express the mind shift in thinking and acting that is considered essential for CLSC management. However, the explicit choice by the SCAs for a follow-up study to jointly develop a plan of action within a ‘safe’ case study environment (connected to a real-world pilot) expresses attainment of situational maturity.

### Situational Maturity and Mental Maturity

Transition theory [e.g. 11, 38] argues that for transitions to happen, double-loop learning processes are required. However, the literature hardly provides answers to the question how these processes can be stimulated. Transition theory literature argues that double-loop learning is unlikely to happen unless exceptional circumstances occur (e.g. a crisis or new legal requirements) [7, 39]. This suggests double-loop learning to be dependent upon external pressure. However, this study shows that double-loop learning can also occur in well-organized projects that explicitly aim to achieve capabilities that are considered necessary for making a transition. The case study showed that the systematic maturity capability approach offers a valuable solution. It is therefore *hypothesized* that by focusing, in each CLSC project, on achieving capabilities to deal with deep uncertainty and dynamic complexity—aimed at reaching situational maturity—the involved SCAs will gradually internalize the different capabilities and develop mental maturity.

Both the theoretical framing and empirical application of the approach underline the importance of distinguishing between double-loop learning in relation to ‘situational maturity’ versus ‘mental maturity’. Two examples may illustrate this in relation to the described case study. First, the systematic capability maturity approach triggered multiple SCAs to think more multidimensionally along the process, however, did not cause the SCAs to systematically think and act more multidimensionally beyond the case study environment. Hence, double-loop learning has taken place, but only on a situational level, i.e. situational maturity has been achieved. Second, since the group discussion on the CLDs, SCA1 identified new feedback loops in relation to closed-loop (timber) construction, which indicates a step forward in the process of mental maturity. In the theoretical framework of this study, the expectation was formulated that situational maturity in different contexts can function as a steppingstone towards reaching mental maturity. Since the present case study did not allow for a definitive conclusion on this issue, it remains a *hypothesis* to be further investigated in future research.

The elaborated capability maturity approach assumes that if all SCAs relevant for making a transition towards CLSC management have internalized all capabilities, mature CLSC management will result. However, this study showed that some SCAs gained capabilities, while others struggled to gain those same capabilities. Hence, it is *hypothesized* that if this asymmetry continues to develop and some SCAs move from situational maturity to mental maturity whereas others stay behind, it will be very difficult in practice to realize mature CLSC management at the level of the supply chain.

### Enriching Knowledge on Content

Content knowledge in this case was enriched using a variety of analytical tools. Regarding the details of the applied toolkit, the study notably triggered doubts about the added value of the ESDMA, at least with respect to how it is practised at present. ESDMA is supposed to support actors in decision-making under deep uncertainty and dynamic complexity. However, Kwakkel and Pruyt [40] argue that it is important to develop and use more effective visualization tools (e.g. (info) graphics) for the presentation of ESDMA results. This study also showed that the presently applied visualisation tools (see Fig. 6) appear insufficiently supportive for the SCAs. This observation reflects the constant need for clarification of differences between the concepts of probability, possibility, and plausibility. In the various discussions among the SCAs, the boundaries between these concepts sometimes got blurred, whereas the implications for decision-making can be significant. A solution would be to involve the actors more actively in performing certain analyses, such as executing ESDMA. Future research should investigate whether such actor involvement indeed leads to a better understanding of, and commitment to, the different dynamic models’ behaviours and related uncertainties. Greater involvement can be achieved by developing a graphical user interface for the modelling and simulation software behind ESDMA.

## Conclusion and Limitations

This study focused on the question how a systematic capability maturity approach could affect the attainment of higher levels of maturity by SCAs who prepare for implementing CLSC management. The focus in this study was on the joint development of CLSC goals. The underlying idea was that maturity growth is related to double-loop learning, notably on the issue of dealing with deep uncertainty and dynamic complexity. The motive for the study is that scientific literature on CLSC management has argued that the lack of capabilities to grasp these phenomena often constitute an obstacle in the transition process.

The facilitated case study showed that involved SCAs indeed gained maturity in grasping the complexity and uncertainty of the supply chain transition, but that not all SCAs have yet reached the capabilities of the Maturity Stages 1 and 2 as defined in the applied methodology. Obvious signs of growth in situational maturity could be observed but only few signs of growth in mental maturity. Hence, the study provides evidence that following a structured participatory process seems helpful in changing the actors’ mind-set and enabling them to indeed move on in the direction of CLSC management under deep uncertainty and dynamic complexity. It is evident that, because this conclusion is based on only one intensive case study, more empirical evidence needs to be gained before more robust conclusions can be drawn about the added value of the systematic capability maturity approach.

The study has limitations. First, there is a potential problem of bias in data collection and analysis due to the facilitating role of the researchers, including structuring the process, executing informative analyses, and making observations. To limit this bias, a priori coding and evaluative interviews were used. Both methods reduce the probability that the researchers only focus on data that confirm the suitability of the applied approach. Second, only parts of the developed system dynamics models were discussed with experts in that domain. A thorough debate with experts can be helpful to limit biases. Specifically, such discussions could focus on certain parameters and variables for which little or no literature is available (e.g. quality, volume, and price of dismantled timber), conversion factors and model structures. Third, because of the COVID-19 pandemic, the study has had a longer lead time than initially planned. On the one hand, it might have been better for the learning effect of the involved SCAs if the research would have had a shorter lead time (less time to return to old habits and less time needed to recall the progress made in the previous steps in the case), yet on the other hand, the longer lead time may also have made a positive contribution to learning since it provided more time to (re)think about information and decisions.

## Data Availability

Data is registered and stored in compliance with the general rules for scientific data management of the Radboud University Nijmegen.
